# The effect of disorder in multi-component covalent organic frameworks†

**DOI:** 10.1039/d3cc01111a

**Published:** 2023-05-05

**Authors:** Emma H. Wolpert, Andrew Tarzia, Kim E. Jelfs

**Affiliations:** a Department of Chemistry, Imperial College London, Molecular Sciences Research Hub White City Campus, Wood Lane London W12 0BZ UK e.wolpert@imperial.ac.uk k.jelfs@imperial.ac.uk +44 20759 43438; b Department of Applied Science and Technology, Politecnico di Torino Corso Duca degli Abruzzi 24 Torino 10129 Italy

## Abstract

We examined the effect of two different types of linker distribution—random or correlated distribution—on the pore size and shape within single-layers of three multi-component COFs. We reveal a relationship between linker distribution and the porosity of COF solid solutions. The methods presented in this paper are generalisable and could be used in further studies to examine the properties of disordered framework materials.

## Abstract 2

We examined the effect of two different types of linker distribution—random or correlated distribution—on the pore size and shape within single-layers of three multi-component COFs.

## Graphical abstract

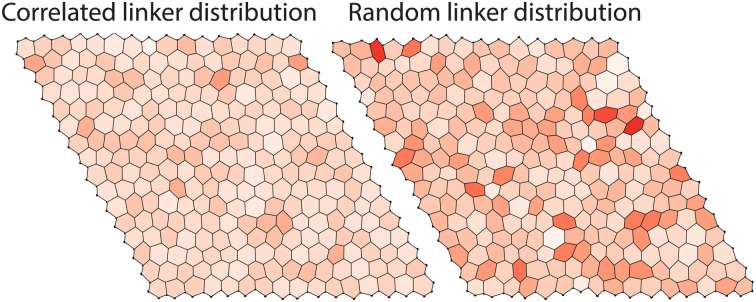

2D covalent organic frameworks (COFs) have attracted considerable attention as they form 1D pore channels with columnar π stacks, resulting in high surface areas and tunable optical, electrical, and photoelectric properties.^1^ They are synthesised by combining two planar monomers, and by virtue of the near endless number of organic monomers, COFs have become a powerful platform for structural design, forming thermally stable, light-weight compounds with a wide range of applications, such as in catalysis,^2^ sensors,^3^ electronics,^4^ and energy storage and conversion.^5^ The emergence of the functional properties of 2D COFs arises from the interplay of the pore shape, the pore size, and the pore wall.^6^ These factors are a consequence of the monomers chosen to form the 2D framework and reticular design principles are often implemented to engineer the pores.

To introduce another level of functionality into COFs, the number of monomers can be increased. For example, introducing two types of linkers: a π-donor and a π-acceptor, creates arrays of donor and acceptor π-columns, facilitating intercolumnar interactions resulting in favourable conductivity.^7^ For most multi-component systems, the linkers order on the lattice, lowering the COFs symmetry.^7–9^ However, recently, Li *et al.*^10^ synthesised a 2D hexagonal COF solid solution where the symmetry of the COF is not lowered on changing linker composition. Therefore, the linker distribution is disordered within the structure. Forming COF solid solutions—homogenous mixtures of multi-component materials with a single crystal structure—unlocks the ability to tune the COFs properties by varying the stoichiometry of the linkers and balancing the monomers’ intrinsic properties.

Although Li *et al.*^10^ showed that the distribution of the linkers is disordered, the arrangement of the linkers may not be random due to correlations between the linker positions. This can occur when there is a chemical reason for linkers of the same type to avoid being near each other *e.g.* due to differences in electronegativity or to minimise the strain introduced from different length linkers. For some topologies with a 1 : 1 ratio of two different linkers, this scenario leads to an ordered arrangement of the linkers (*e.g.* on a square lattice the linkers alternate around each node), but for others, such as hexagonal COFs (Fig. 1(a)), linkers of the same type are forced to neighbour one another as the nodes are connected to an uneven number of linkers. It is this type of frustration that leads to the formation of correlated disorder, as there are chemical rules guiding local configurations (*e.g.* no node will have three of the same type of linker around it (Fig. 1(b))), but these “rules” give no long-range order as there is an equal probability of each node connecting to two linkers of either type (Fig. 1(c)). Correlated disorder is widely reported in materials,^11^ such as in the packing arrangement of proteins,^12^ electronic structure of topological insulators,^13^ and in the spin structure of magnetic materials.^14,15^ But how does having a correlated linker distribution in COFs affect the properties of the material, and can it be used advantageously in pore engineering?

**Fig. 1:**
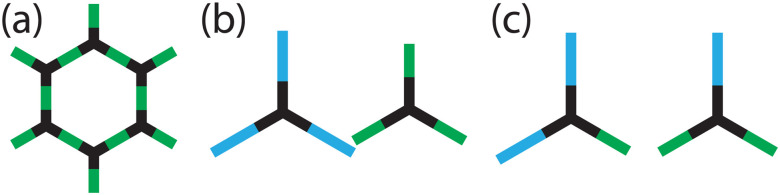
(a) Hexagonal COF formed by combining ditopic linkers (green) and tritopic nodes (black). Distributions around each node that are (b) energetically unfavourable and (c) energetically favoured when linkers prefer to have neighbours of different types.

In this paper, we analyse the effect of monomer distribution on the pore size and shape of COFs formed from the condensation of 1,3,5-tris(4-aminophenyl) benzene (TAPB) with a 1 : 1 mixture of two of the three dialdehydes: terephthalaldehyde (PDA), 4,4′-biphenyldicarbaldehyde (BDA), or [1,1′ : 4′,1′′-terphenyl]-4,4′′-dicarbaldehyde (TDA) which have one, two, or three benzene rings respectively (Fig. 2). These form the three mixed linker COFs: TAPB-(PDA_0.5_BDA_0.5_), TAPB-(BDA_0.5_TDA_0.5_), and TAPB-(PDA_0.5_TDA_0.5_). We find that the different linker lengths have a negligible effect on the pore size and shape when the ratio between the linker lengths is small (≈5 : 6), and a more significant effect for larger differences (≈5 : 7). These findings may have implications in using COF solid solutions as membranes because the distribution of the linkers affects the extent to which the pore size and shape can be selectively tuned for.^16,17^ Moreover, the methods reported in this paper provide a route to produce atomistic models of disordered COFs using our software, *stk*,^18^ that can be used to study the effect of disorder on other COF properties.

**Fig. 2:**
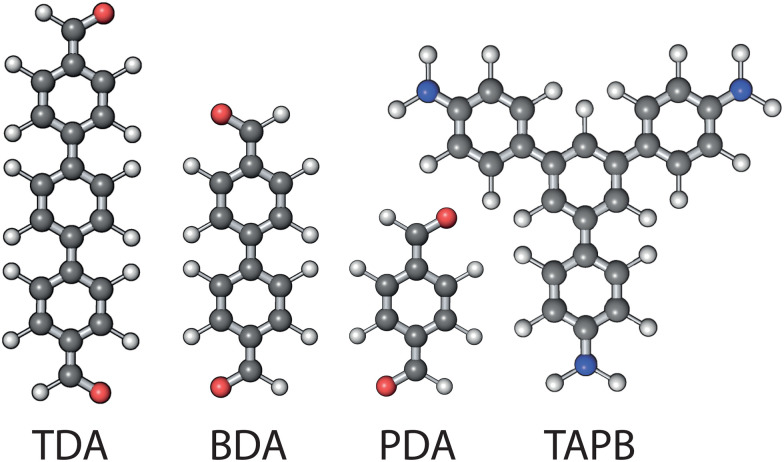
The monomers used in the formation of the disordered COFs. C, O, N, and H atoms are shown in black, red, blue, and white respectively.

We implemented Monte Carlo (MC) simulations to produce COFs with random and correlated linker distributions. A starting configuration corresponding to a 16 × 16 supercell of the underlying kagome unit cell (Fig. S1, ESI†) was decorated randomly with states *S*_*i*_ = ±1 representing the linkers such that when *S* = +1(−1) the site was populated with a long(short) linker. The MC simulations used the Metropolis algorithm with periodic boundary conditions where sites were randomly selected and inverted. The energy of each move was calculated according to the Hamiltonian1
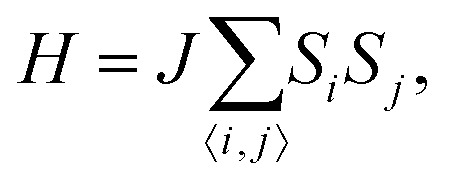
where *J* is the interaction parameter and 〈*i*,*j*〉 denotes a sum over nearest neighbour sites *i*,*j*. *J* > 0 such that it is energetically preferable for the linkers around each node to be different. To produce configurations with random (correlated) distributions of linkers, the simulations were performed at high (low) temperatures where *T*/*J* = 25(0.25). Although setting the temperature instead of cooling the simulations can result in a metastable state, the configurations from the low-temperature simulations were examined to ensure there were no sites with three of one type of linker around each node. Each MC simulation ran for 100 000 × *n*^3^ steps where *n* is the size of the supercell and was repeated 5 times to average the results.

The outputted structure from the MC simulations were geometry optimised to minimise the strain to ensure a sensible representation of the COF structure. As the outputted configuration for a 16 × 16 supercell contained at least 36 000 atoms, geometry optimisation of the atomistic structure was deemed too computationally expensive. Instead, the structure was coarse-grained, placing a single atom at the centre of mass of each monomer whose atom type represented the monomer's identity (Fig. S2(a and b), ESI†). This coarse-grained structure was geometry optimised using GULP,^19^ with harmonic potentials between the “atoms” set to represent the different monomer lengths (Section S3, ESI†). After optimisation, the structure was converted to the atomistic COF using *stk*^18^ (Fig. S2(c and d), ESI†). To validate our methodology, we compared our results to a 4 × 4 atomistic supercell which was relaxed further in GULP using the UFF force field.^20^ Due to the smaller number of pores per configuration (16 *vs*. 256 for 16 × 16 supercells), we averaged the results over 10 configurations instead of 5. This produced comparable results (Section S5, ESI†), validating the use of the coarse-grained model.

For each COF, the pore size distribution was determined using Zeo++^21^ with the *c* lattice parameter set to 5 Å to ensure that only the size of the pores within the layer was measured. The pore size distributions were calculated using the high accuracy flag with 50 000 MC samples, a probe diameter of 1.2 Å, and was plotted using kernel density estimation in Seaborn: bandwidth method Scott's, adjust is 1. As the pore size distribution overlooks the nuances in the structure that arise from changes in the shape of the pore due to different linker combinations, we measured “how hexagonal” the pores were by calculating the similarity of the pore shape to a perfect hexagon, HP-6, using the SHAPE software.^22,23^ Given a coarse-grained model, we extracted the atoms representing the monomers which defined each hexagon (*i.e.* TAPB), using the atomic simulation environment.^24^ For the 4 × 4 atomistic models which were relaxed further, we used the cheminformatics Python package, RDKit,^25^ to convert the TAPB molecule to a single atom at its centroid, where each centroid is bonded to its neighbouring TAPB centroid. The HP-6 measure was calculated for each six-member ring, where the closer HP-6 is to 0, the more hexagonal the pore is. Code for converting the coarse-grained models to atomistic structures using *stk* and performing shape analysis is open-sourced here https://github.com/andrewtarzia/cg-2d-cofs.

The MC simulations only considered the distribution of linkers within a single-layer of the COF, and as such, our results exclude the effect of stacking behaviour on porosity. As it has been reported that both the TAPB-PDA and TAPB-BDA COFs, as well as the solid solutions formed by them, have eclipsed stacking, we expect the single-layer pore behaviour to capture most of the features of interest.^10,26,27^ However, we have included a discussion of how the different pore size and shape distributions may affect or disrupt this eclipsed stacking.

Our results (Fig. 3) show that the pore size distribution is broader for larger differences in the linker lengths. The average pore size decreases between the correlated distribution and randomly distributed linkers, the magnitude of which follows the same trend as the ratio of linker lengths: PDA/TDA > PDA/BDA > BDA/TDA (Fig. 4(a)). This effect is more marked for PDA/TDA, where the change in the mean pore size is ≈ 3 times greater than for the other COFs. For all structures, the pore size distribution is broader when the linkers are distributed randomly rather than in a correlated fashion, evidenced by the increase in the standard deviation (Fig. 4(a)). For the PDA/BDA and BDA/TDA COFs, this broadening of the pore distribution is small, likely having a negligible effect on the COF properties, whereas the broadening of the pore size distribution is more pronounced for the PDA/TDA structure which may effect the properties of the COF. For example, a broader distribution could lead to less selectivity in molecular separation, hindering the ability to fine-tune the pore size based on linker ratio. As the pore size distribution is broad for all of the COFs and types of linker distribution, our findings suggest that solid solutions would be less selective membranes than their ordered counterparts.

**Fig. 3:**
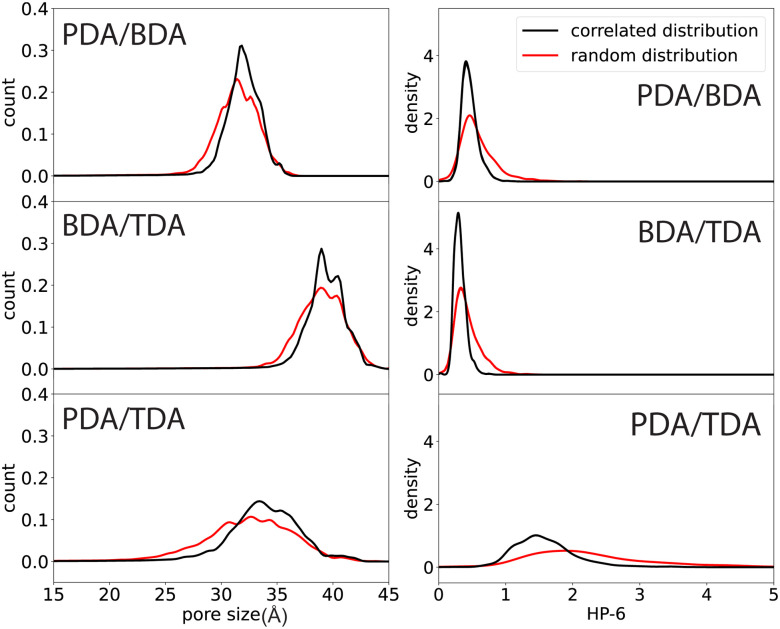
Pore size (left) and shape (right) distribution for the different linker combinations.

**Fig. 4:**
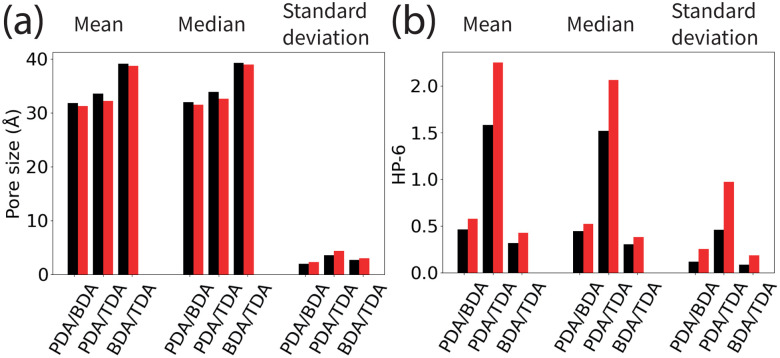
The mean, median, and standard deviation of (a) the pore sizes in the COFs and (b) HP-6 for COFs. Black/red bars correspond to the correlated/random distribution of linkers in the structures.

For the pore shape, the anisotropy increases with the ratio of linker lengths: BDA/TDA < PDA/BDA < PDA/TDA, evidenced by the increased average value of HP-6 (Fig. 3, 4(b) and 5). Similar to the pore size distribution, the pore shape distribution is broader for randomly distributed linkers than correlated structures, and is broadest for PDA/TDA. This is evidenced by the standard deviation of randomly distributed COFs being more than double that of correlated cases, and by the standard deviation of PDA/TDA being more than three times greater than BDA/TDA or PDA/BDA (Fig. 4(b)). The difference in the average value of HP-6 between the correlated and random distribution of linkers follows the trend of increasing ratio of linker lengths, and is most pronounced for PDA/TDA, where the mean value of the pore shape is ≈ 6 times greater than for BDA/TDA or PDA/BDA. Thus, the different distribution of the linkers has the largest effect for PDA/TDA, likely changing its utility in molecular separations. Given the size of the pores, the COF's applications would be most likely be in the separation of large biomolecules which are often anisotropic and as such the anisotropy of the pore shape can have a marked effect on the selectivity.

**Fig. 5:**
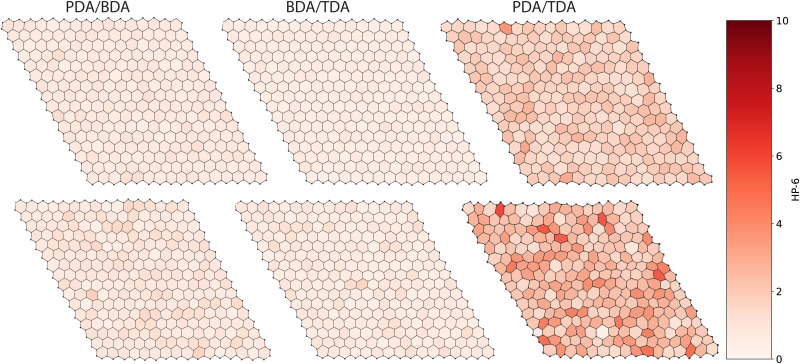
Outlines of representative COF structures produced from the MC simulations with a correlated distribution of linkers (top) and randomly distributed linkers (bottom) for each linker combination. Hexagons within the structure are coloured by their similarity to a perfect hexagon, HP-6.

The anisotropic pore size and shape will effect the COFs layer alignment, impacting the material properties. As the monomers polymerise to form 2D sheets before stacking,^28^ the distribution between layers is likely to be uncorrelated. Therefore, the broad pore size distribution and anisotropic pore shapes will likely lead to worse alignment between the layers compared to ordered COFs. This may affect the COF's utility in molecular separations as the worse the alignment, the more staggered the stacking, decreasing the width of the pore channels. The extent of misalignment will likely be more pronounced for COFs with randomly distributed linkers due to the broader pore size distribution, possibly making them suitable for chromatography applications similar to gel permeation columns. In these systems, the misalignment between layers can lead to smaller molecules becoming entangled in the structure, having a longer path length than larger molecules, separating the two. A caveat to this is that a greater extent of misalignment likely results in weaker interlayer interactions and thus significantly more stacking disorder than in the correlated disordered case. Since stacking disorder results in less control over the size and shape of the pore channels, one would expect this to hinder the COFs application in molecular separations. However, 2D COFs with stacking disorder have recently proved useful in the separation of benzene and cyclohexane.^29^ On the other hand, the weaker interactions from the misalignment between layers also results in a higher propensity to form polycrystalline structures, hindering selectivity.^29^

In summary, we have studied how linker distribution affects the properties of COF solid solutions. We show that the pore size and shape distribution is broader for randomly distributed linkers rather than for correlated distributions. For small ratios of linker lengths (≈5 : 6), this effect is negligible, but for a larger differences (≈5 : 7) this effect is larger, which may affect the application of COFs in membrane separation. Developing an understanding of the stacking behaviour in COF solid solutions will enable more accurate determination of the effect of disorder on the properties. However, layered COFs are notorious for containing stacking disorder, making studies on the structural behaviour of ordered COFs difficult, let alone structurally disordered COFs.^30^

Through this work we have presented a computationally inexpensive method of creating structural models of disordered COFs. Outside of porosity, the distribution of linkers in COFs can impact other properties such as the electronic and optical properties. The process outlined in this paper can help create atomistic models for further property prediction of disordered COFs, elucidating a disorder/property relationship.

K.E.J acknowledges the Royal Society for a University Research Fellowship and Enhancement Award and the ERC through Agreement No. 758370 (ERC-StG-PE5- CoMMaD). We thank Becky Greenaway and Annabel Basford for helpful discussions.

## Conflicts of interest

There are no conflicts to declare.

## Supplementary Material

CC-059-D3CC01111A-s001
