# Supplementary material for: The effect of disorder in multi-component covalent organic frameworks
Source: Chem Commun (Camb). 2023 May 5;59(45):6909–12. doi: 10.1039/d3cc01111a (PMC10234295; doi:10.1039/d3cc01111a)
Supplement: CC-059-D3CC01111A-s001 [file CC-059-D3CC01111A-s001.pdf]

Electronic Supplementary Material (ESI) for ChemComm.
This journal is © The Royal Society of Chemistry 2023

Supporting Information: The effect of disorder in multi-component covalent organic frameworks

Emma H. Wolpert,[*,†] Andrew Tarzia,[‡,†] and Kim E. Jelfs[*,†]

†*Department of Chemistry, Imperial College London, Molecular Sciences Research Hub, White City Campus, Wood Lane, London, W12 0BZ, UK*

‡*Department of Applied Science and Technology, Politecnico di Torino, Corso Duca degli Abruzzi 24, 10129 Torino, Italy*

E-mail: e.wolpert@imperial.ac.uk; k.jelfs@imperial.ac.uk

Contents

1 **Mapping the COF to a kagome lattice** **S-3**

2 **Relation of atomistic structure to coarse-grained structure** **S-3**

3 **Geometry optimisation of coarse-grained models** **S-4**

4 **HP-6 values** **S-5**

5 **Atomistic 4×4 simulations** **S-9**

1 Mapping the COF to a kagome lattice

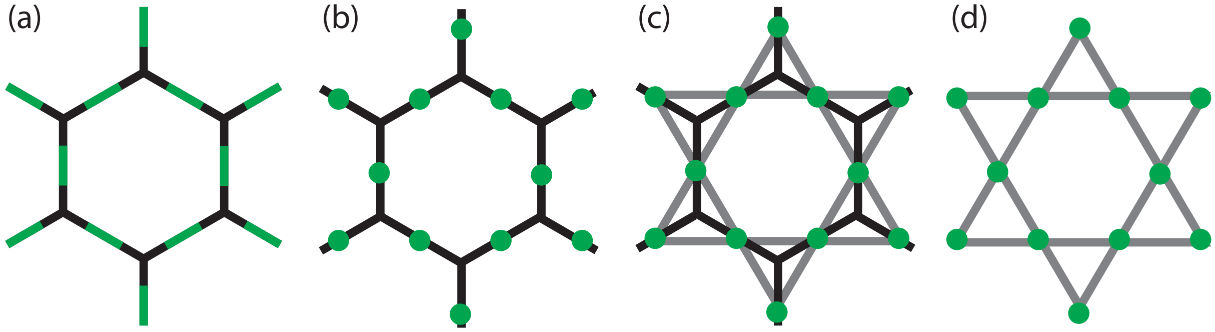

Figure S1: Mapping between the hexagonal COF structure to the kagome lattice. (a) a 2D hexagonal COF with a tritopic node (black) and a ditopic linker (green). (b) The linkers are represented as green spheres ontop of the underlying hexagonal lattice (black). (c) An overlay of the kagome lattice (grey) on top of the hexagonal lattice. (d) The kagome lattice where the linkers now sit on the lattice nodes and each site (green sphere) has a state associated with it representing the linker type.

2 Relation of atomistic structure to coarse-grained structure

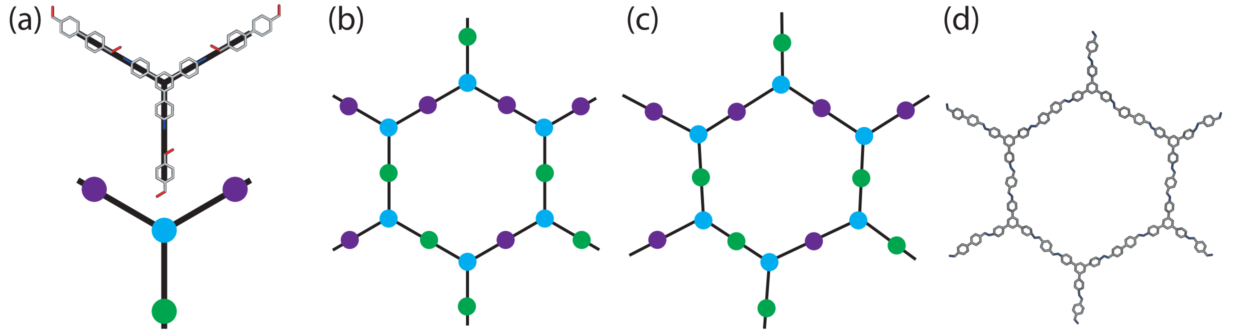

Figure S2: (a) Mapping of atomistic linkers on the hexagonal lattice to different atom types which represent each of the linkers. Here the blue, purple, and green atom represent the TAPB, BDA, and PDA molecule respectively. (b) These atoms were then placed on the lattice according to the results of the Monte Carlo simulations. This allowed us to run computationally inexpensive geometry optimisations and convert the optimised (c) coarse-grained structure back to the (d) corresponding atomistic structure.

3 Geometry optimisation of coarse-grained models

Table S1: Equilibrium bond lengths used in geometry optimisations

Linkers	Short bond length Å	Long bond length Å	Difference in bond length Å
PDA/BDA	10.396	12.648	2.252
PDA/TDA	10.396	14.767	4.371
BDA/TDA	12.648	14.767	2.119

The geometry optimisations of the coarse-grained models were performed using GULP under constant pressure. Harmonic bonds were created between the atoms representing the linkers and nodes with the bond strength $k = 100\,\text{eV}\text{Å}^{-2}$. Three body potentials were also implemented for each atom as set out in Table S2.

Table S2: Three-body potentials used in GULP optimisations

Three-body	Angle °	Bond strength eVÅ^{-2}
TAPB-Linker-TAPB	180	300
Linker-TAPB-Linker	120	200

The size of each super cell was chosen for the starting configuration by averaging the supercell sizes from the single linker structures, giving the a, b cell parameters of 640 Å, 704 Å, and 768 Å for PDA/BDA, PDA/TDA, and BDA/TDA respectively. The c-axis parameter for each of the structures was set to 100 Å. For the 4×4 simulations, the cell parameters were 160 Å, 176 Å, and 192 Å for PDA/BDA, PDA/TDA, and BDA/TDA respectively.

4 HP-6 values

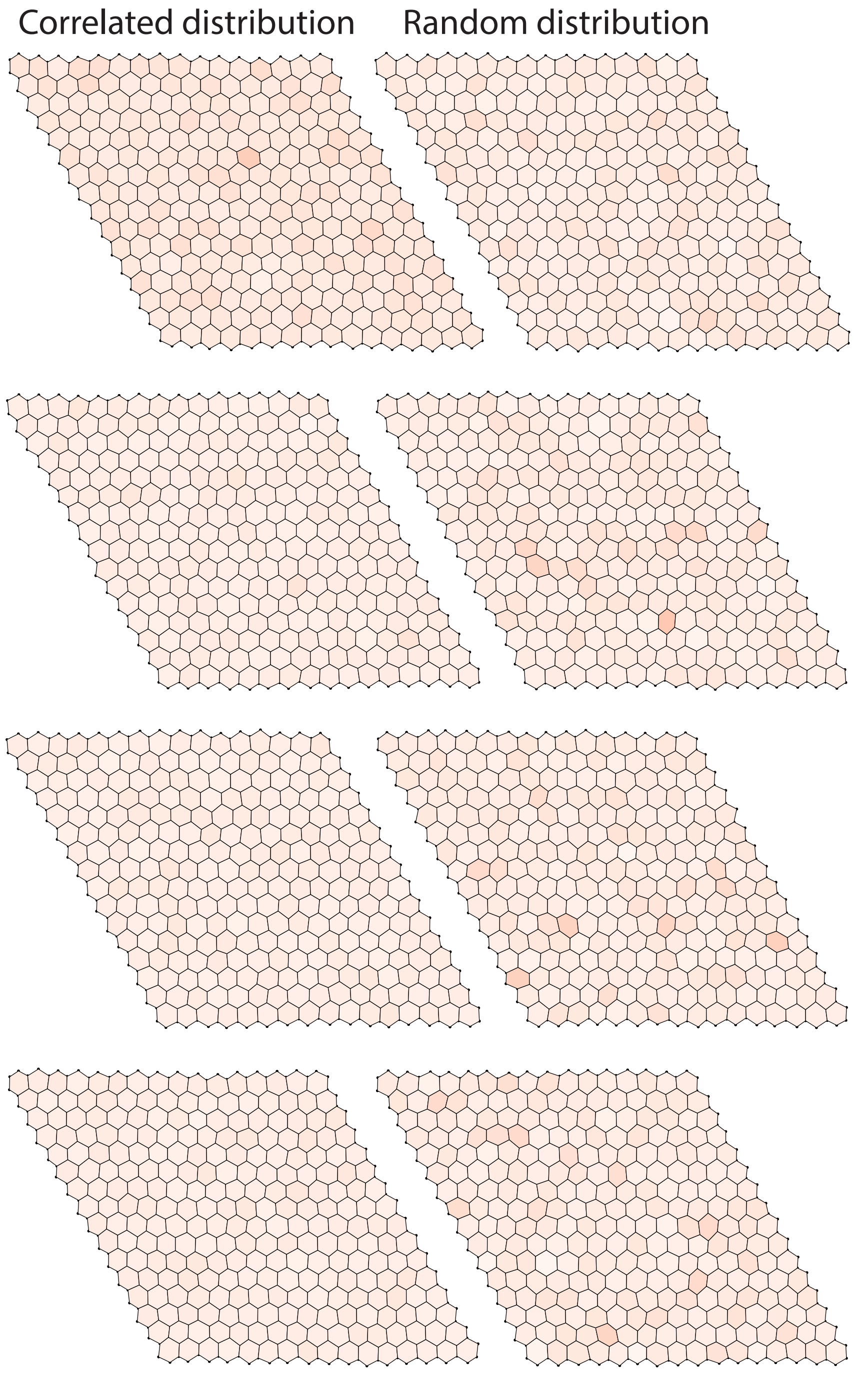

Figure S3: HP-6 results for the 4 simulations not shown in the main paper for PDA/BDA COFs. The outline of the COF structures from each simulation with a correlated distribution of linkers (left) and randomly distributed linkers (right). Each hexagon within the structure is coloured by its value of HP-6, i.e. a measure of how similar the pore is to being a perfect hexagon, and the color scale is shown in the main paper.

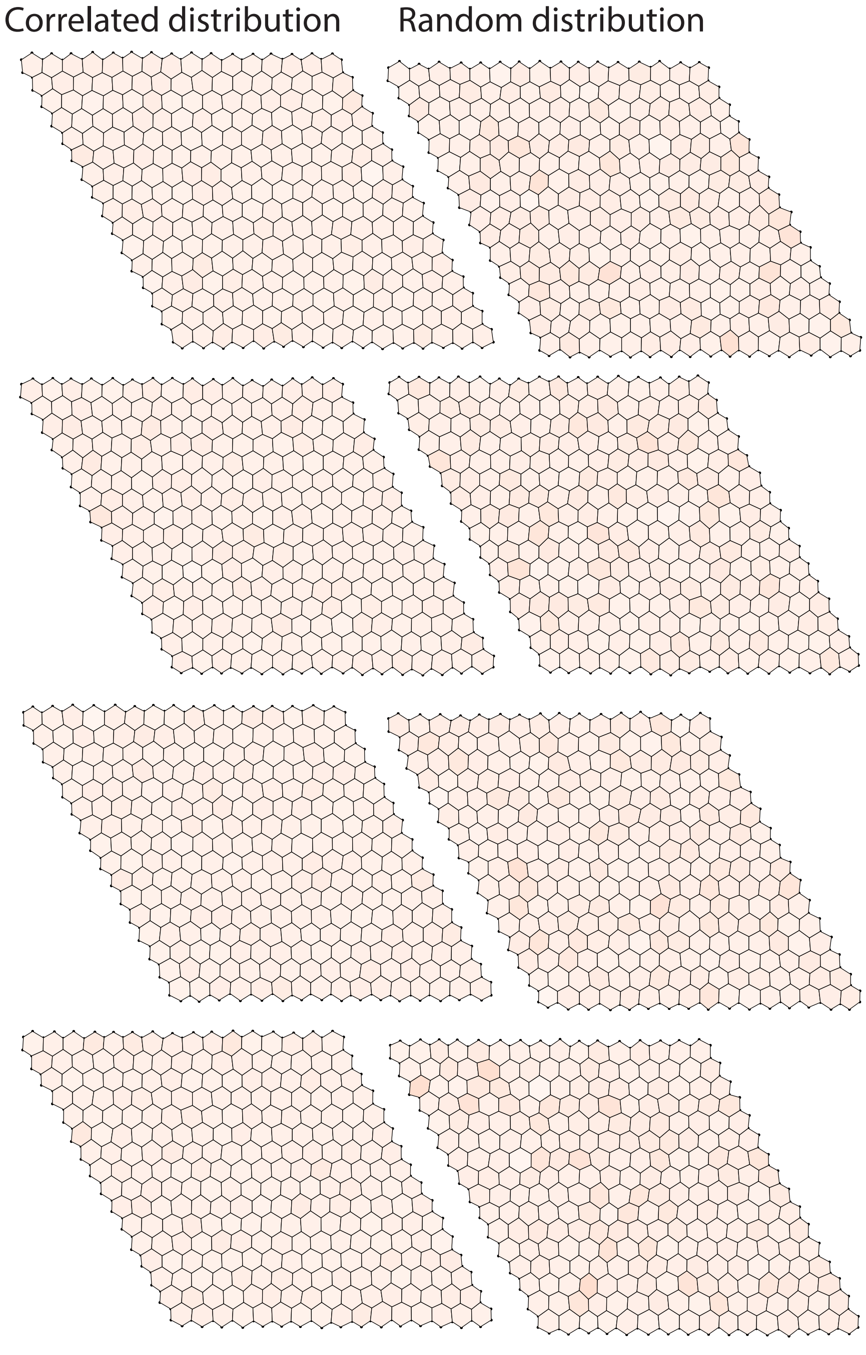

Figure S4: HP-6 results for the 4 simulations not shown in the main paper for BDA/TDA COFs. The outline of the COF structures from each simulation with a correlated distribution of linkers (left) and randomly distributed linkers (right). Each hexagon within the structure is coloured by its value of HP-6, i.e. a measure of how similar the pore is to being a perfect hexagon, and the color scale is shown in the main paper.

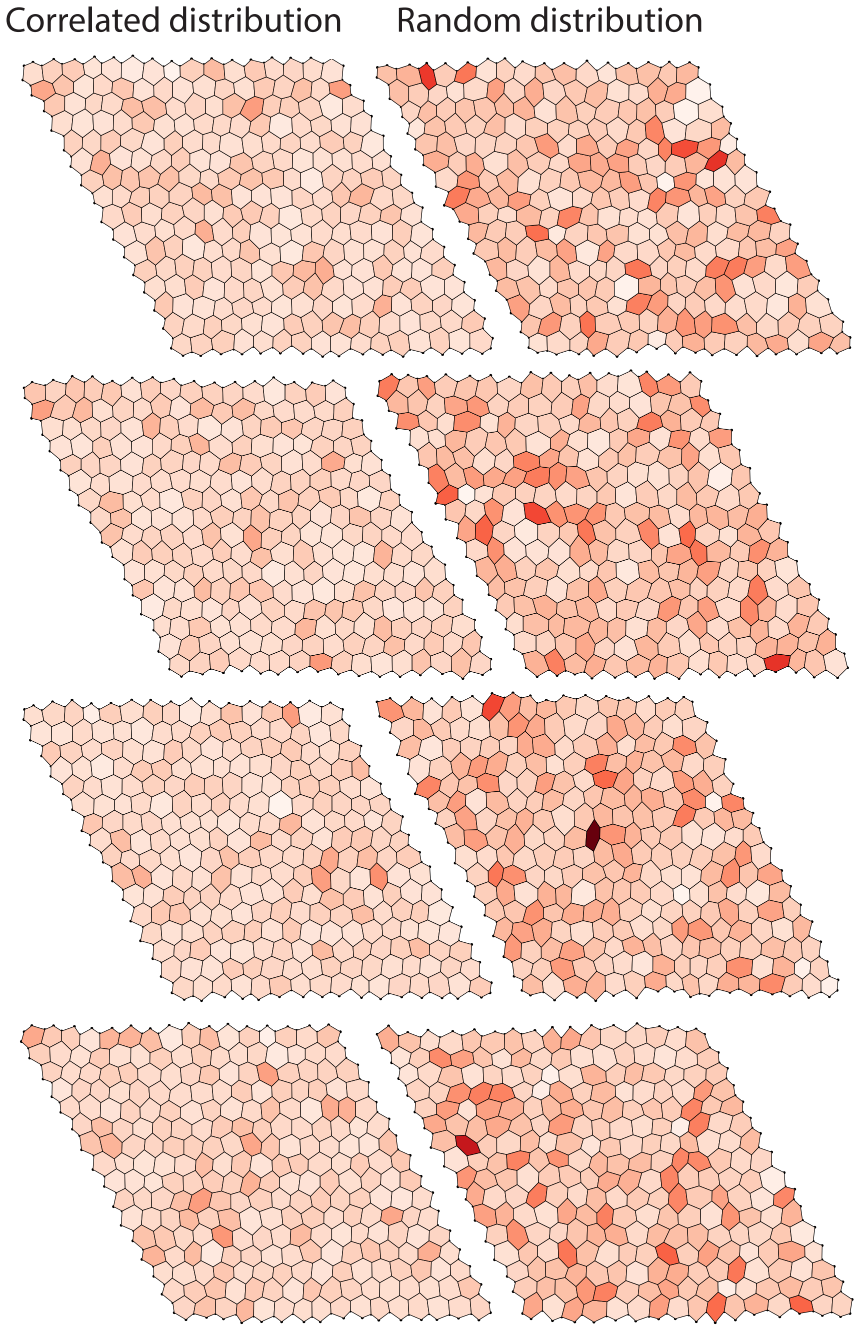

Figure S5: HP-6 results for the 4 simulations not shown in the main paper for PDA/TDA COFs. The outline of the COF structures from each simulation with a correlated distribution of linkers (left) and randomly distributed linkers (right). Each hexagon within the structure is coloured by its value of HP-6, i.e. a measure of how similar the pore is to being a perfect hexagon, and the color scale is shown in the main paper.

5 Atomistic 4×4 simulations

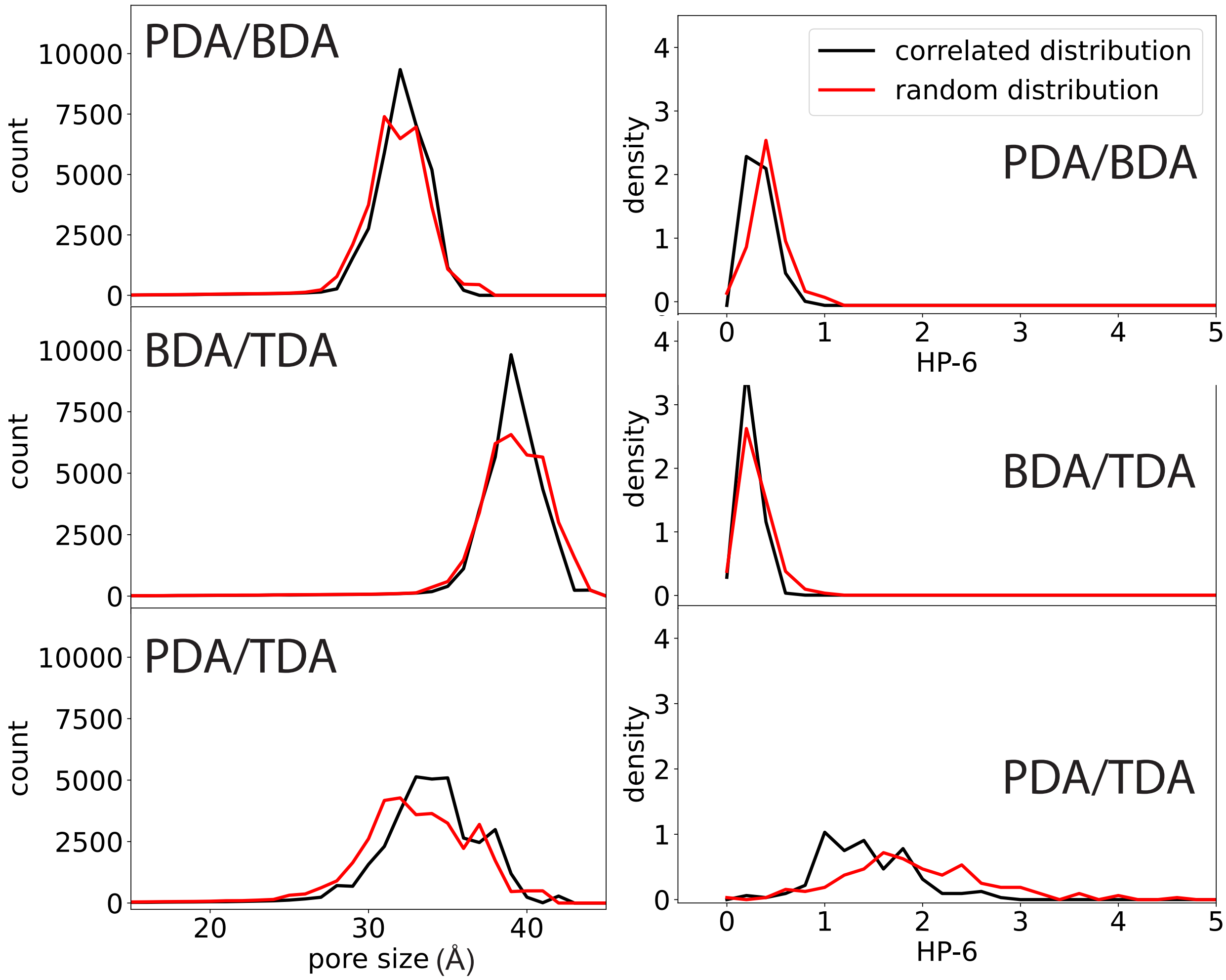

Figure S6: Pore size (left) and shape (right) distribution for the different linker combinations for 4×4 supercells which underwent further relaxation with the atomistic structure after the initial coarse grained relaxation.

Table S3: The mean, median, and stand deviation of the pore sizes in COFs where the C and R represent when the linkers are distributed in a correlated disordered fashion and randomly, respectively.

Linkers	Mean Å		Median Å		Standard deviation Å	
	C	R	C	R	C	R
PDA/BDA	32.4	32.2	32.6	32.4	2.1	2.2
PDA/TDA	34.2	33.8	34.3	34.0	3.6	3.8
BDA/TDA	39.3	39.4	39.6	39.6	2.7	2.8

Table S4: The mean, median, and stand deviation of HP-6 for COFs where the C and R represent when the linkers are distributed in a correlated disordered fashion and randomly, respectively.

Linkers	Mean		Median		Standard deviation	
	C	R	C	R	C	R
PDA/BDA	0.439	0.515	0.412	0.501	0.123	0.196
PDA/TDA	1.519	2.013	1.467	1.940	0.460	0.738
BDA/TDA	0.332	0.386	0.314	0.356	0.098	0.158

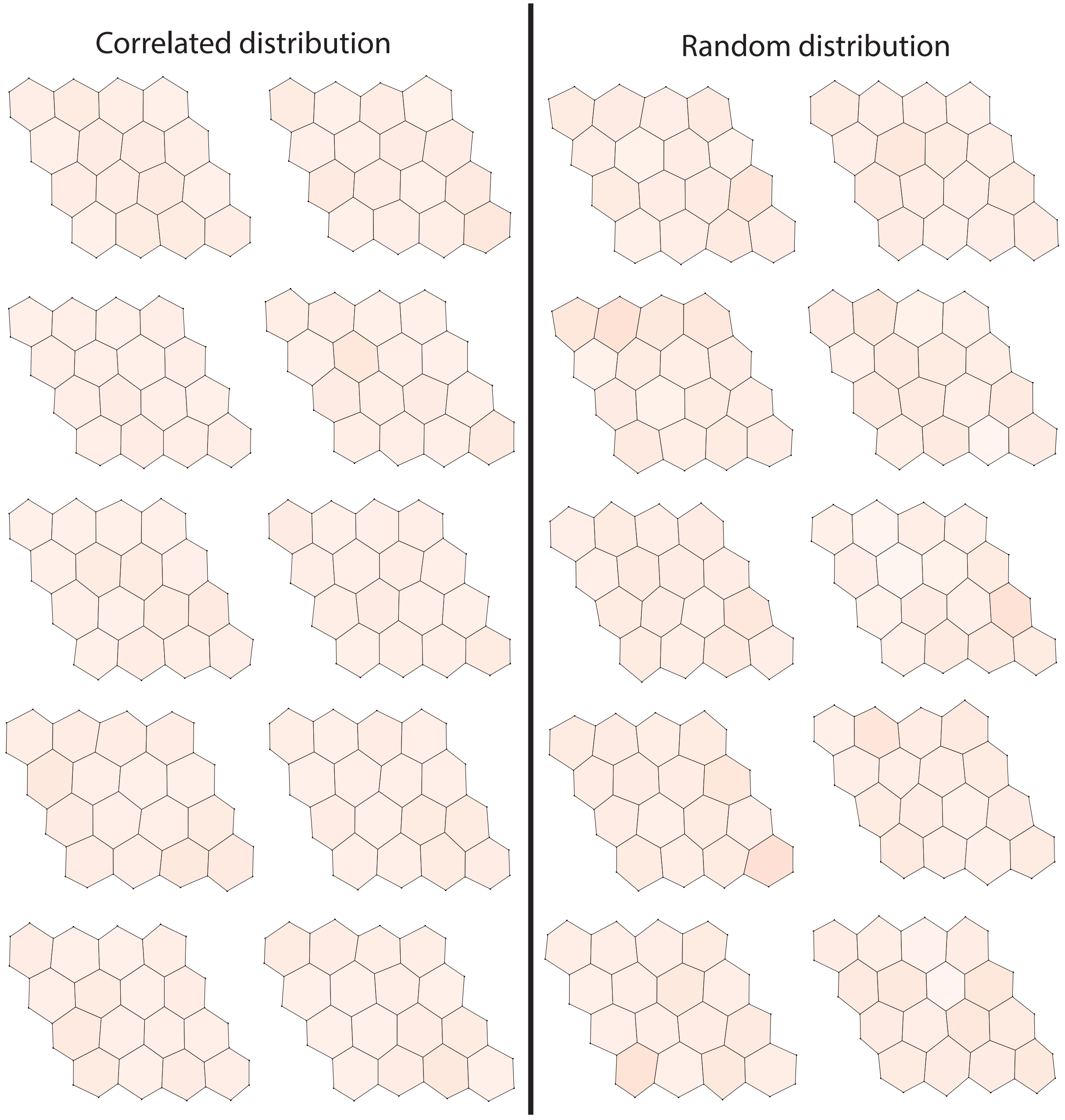

Figure S7: HP-6 results for the 10 4×4 simulations that have undergone atomistic relaxation for PDA/BDA COFs. The outline of the COF structures from each simulation with correlated distributed linkers (left) and randomly distributed linkers (right). Each hexagon within the structure is coloured by its value of HP-6, i.e. a measure of how similar the pore is to being a perfect hexagon, and the color scale is shown in the main paper.

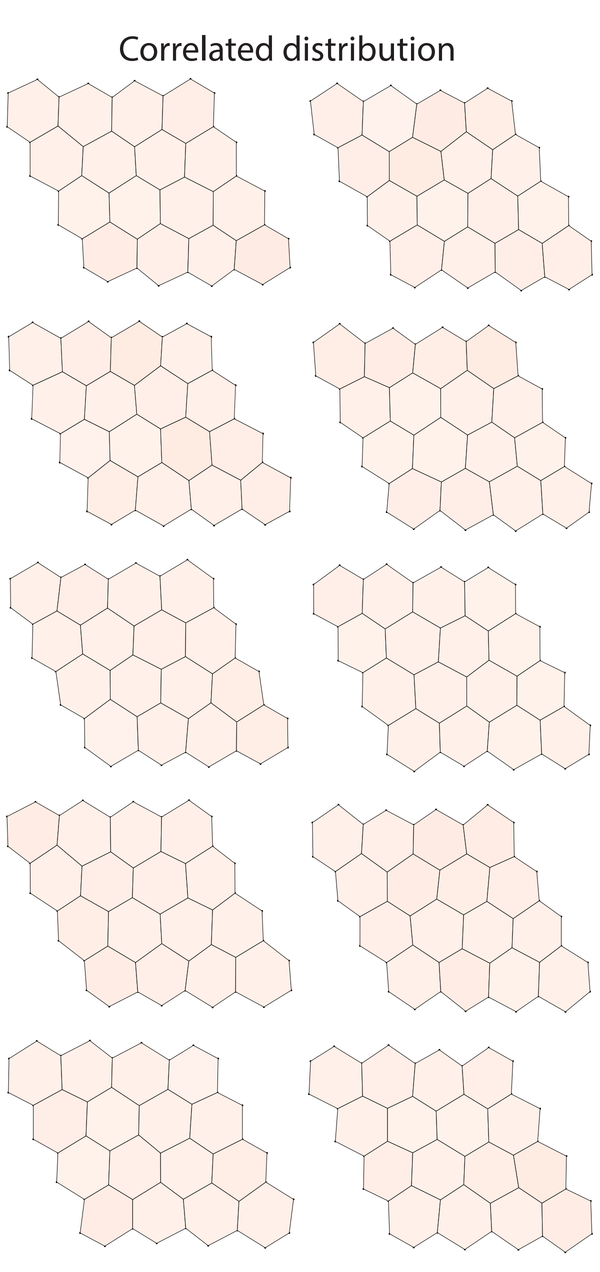

Figure S8: HP-6 results for the 10 4×4 simulations that have undergone atomistic relaxation for BDA/TDA COFs. The outline of the COF structures from each simulation with correlated distributed linkers (left) and randomly distributed linkers (right). Each hexagon within the structure is coloured by its value of HP-6, i.e. a measure of how similar the pore is to being a perfect hexagon, and the color scale is shown in the main paper.

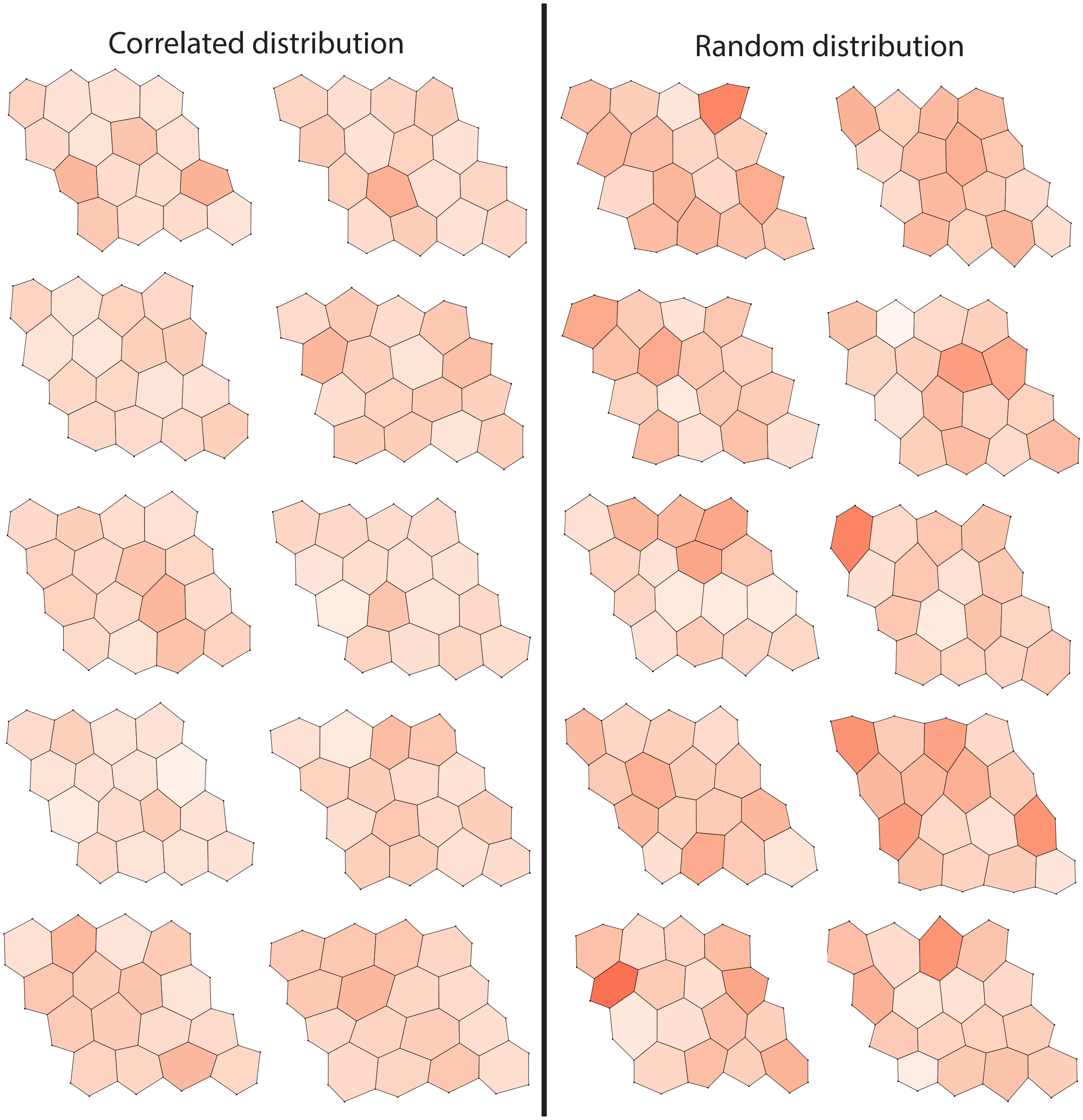

Figure S9: HP-6 results for the 10 4 × 4 simulations that have undergone atomistic relaxation for PDA/TDA COFs. The outline of the COF structures from each simulation with correlated distributed linkers (left) and randomly distributed linkers (right). Each hexagon within the structure is coloured by its value of HP-6, i.e. a measure of how similar the pore is to being a perfect hexagon, and the color scale is shown in the main paper.
